# Pattern reinstatement and attentional control overlap during episodic long-term memory retrieval

**DOI:** 10.1038/s41598-022-14090-4

**Published:** 2022-06-24

**Authors:** Melinda Sabo, Daniel Schneider

**Affiliations:** Leibniz Research Centre for Working Environment and Human Factors, Ardeystraße 67, 44139 Dortmund, Germany

**Keywords:** Attention, Long-term memory, Working memory

## Abstract

Episodic long-term memory (eLTM) retrieval involves the reinstatement of neural patterns from the encoding phase. However, recent evidence suggests that comparable cortical activity patterns can also be linked to attentional control processes on the level of memory representations. The current investigation assesses these two processes independently based on alpha-beta-band activity in the electroencephalogram (EEG). During encoding, subjects were presented with an object on a certain position on the screen and had to imagine it on a new position. In each trial, either the task-irrelevant presentation position or the task-relevant imagination position was lateralized. In the retrieval phase, subjects first made an old/new judgement based on centrally presented objects and then reported the imagination position. Pattern reinstatement should be reflected in similar lateralized alpha-beta activity during encoding and retrieval. Conversely, the influence of attentional control processes during retrieval would be associated with the suppression of alpha-beta power contralateral to the to-be-reported imagination position and with the increase of activity contralateral to the irrelevant presentation position. Our results support this latter pattern. This shows that an experimental differentiation between selective attention and pattern reinstatement processes is necessary when studying the neural basis of eLTM retrieval.

## Introduction

The uniqueness of episodic memory consists in its capacity to allow individuals to consciously re-experience past events (i.e., to do mental time travel) by retrieving details of a particular episode^1^. One central mechanism determining episodic memory retrieval is ecphory. The term was defined by Tulving as a synergistic process through which information stored in the form of memory traces interacts with the retrieval cue to facilitate the recollective experience^2^. Thus, in Tulving’s view, the engram of an event alone is not sufficient unless it is accompanied by a compatible retrieval cue^2^, which prompts a rapid, unconscious reactivation of sensory memory traces^3,4^. Moreover, the retrieval cue seems to not only trigger an early information reactivation, but also the replay of neural patterns from the encoding phase.

Since Tulving’s phenomenological description of ecphory, several studies aimed to explore this phenomenon at a neurophysiological level. The results of these studies suggest that episodic memory retrieval entails a very early “replay” or reinstatement of cortical patterns present during the original event^5,6^, which can happen as early as 100–300 ms after cue presentation^7^. This finding proves to be robust across studies using different brain imaging techniques (e.g., functional magnetic resonance imaging; fMRI^8–10^, EEG^7^, intracranial EEG^11^, magnetoencephalogram^12^) and different types of to-be-remembered stimuli (e.g., words and imagined objects^13^, verbal recall of short movies^5^). Moreover, disruption of this early cortical reinstatement process is suggested to have detrimental effects on episodic memory performance, thus showing that early replay is functionally relevant for episodic memory retrieval^4^.

Yet, a recent study conducted by Sutterer and colleagues^14^ questions the idea that cortical activity underlying pattern reinstatement reflects a single process. In their paper, the authors suggest that alpha oscillations (8–12 Hz) code for spatial locations associated with different objects in the study phase and these patterns re-emerge in the retrieval phase. Nonetheless, they further argue that this activity might as well reflect sustained attention to spatial locations linked to each object. Similarly, Waldhauser and colleagues^4^ reveal that lateralized alpha–beta oscillations track spatial positions in an analogous way during encoding and later retrieval. The authors interpret their results as evidence for pattern reinstatement. However, one cannot exclude the possibility that the underlying alpha activity reflects an overlap between pattern reinstatement and further processes, such as attentional control mechanisms.

A considerable amount of research demonstrates the involvement of alpha oscillations in tracking the focus of attention, both at a perceptual level^15–17^ and within working memory representations^18–22^. The latter finding is especially evident in paradigms, in which the relevance of different items held in working memory changed throughout the task, requiring subjects to switch the focus of attention from one item representation to another. This process of prioritization can, for example, be tracked by lateralized alpha oscillations, showing a contralateral decrease with respect to currently relevant item representations and a contralateral increase corresponding to irrelevant ones^23,24^. Consequently, lateralized alpha activity is not only suggested to play an important role in deploying attention to relevant memory representations, but also in inhibiting irrelevant ones^25–28^. The idea of attentional mechanisms playing an active role in eLTM retrieval is further supported by studies aiming to understand the involvement of the posterior parietal cortex (PPC) in episodic memory retrieval. For instance, it is suggested that activity observed during episodic memory retrieval in the two sub-regions of the PPC, the medial intraparietal sulcus (IPS) and superior parietal lobule, reflects deployment of top-down attention^29^. Similarly, a recent review highlights the importance of the lateral IPS and postcentral gyrus in the manipulation and transformation of information according to the current task goals, a processes argued to occur rather late^30^ and highly likely as the result of attentional mechanisms^29^. These conclusions are also in line with the predictions of the Attention to Memory (AtoM) model^31^, which emphasizes the role of bottom-up and top-down attention in episodic memory retrieval^31^. Accordingly, bottom-up attention captures goal relevant information once this is retrieved by the medial temporal lobe (MTL), while top-down attention is important for maintaining the retrieval goal and for guiding the search for relevant information. The second mechanism becomes especially decisive in cases of poor memories, requiring more effortful memory searches^30,32^.

Taken together, a considerable amount of research confirms the necessity of pattern reinstatement for episodic memory retrieval^7–12^. However, there is also evidence that attention facilitates the retrieval of information from episodic memory^29–31^. As such, it seems plausible that the cortical activity thought to be a marker of pattern reinstatement reflects some further operations, potentially overlapping during retrieval. In this context, it becomes highly unclear whether task-specific modulations of cortical activity previously reported during eLTM retrieval (e.g., alpha oscillations^4,14^) are a neural correlate of pattern reinstatement or also of attentional control processes. We therefore designed an EEG experiment to answer this question and to shed light on the exact role of cortical activity (here measured by topographic changes of alpha and beta oscillations) during eLTM retrieval.

**Figure 1:**
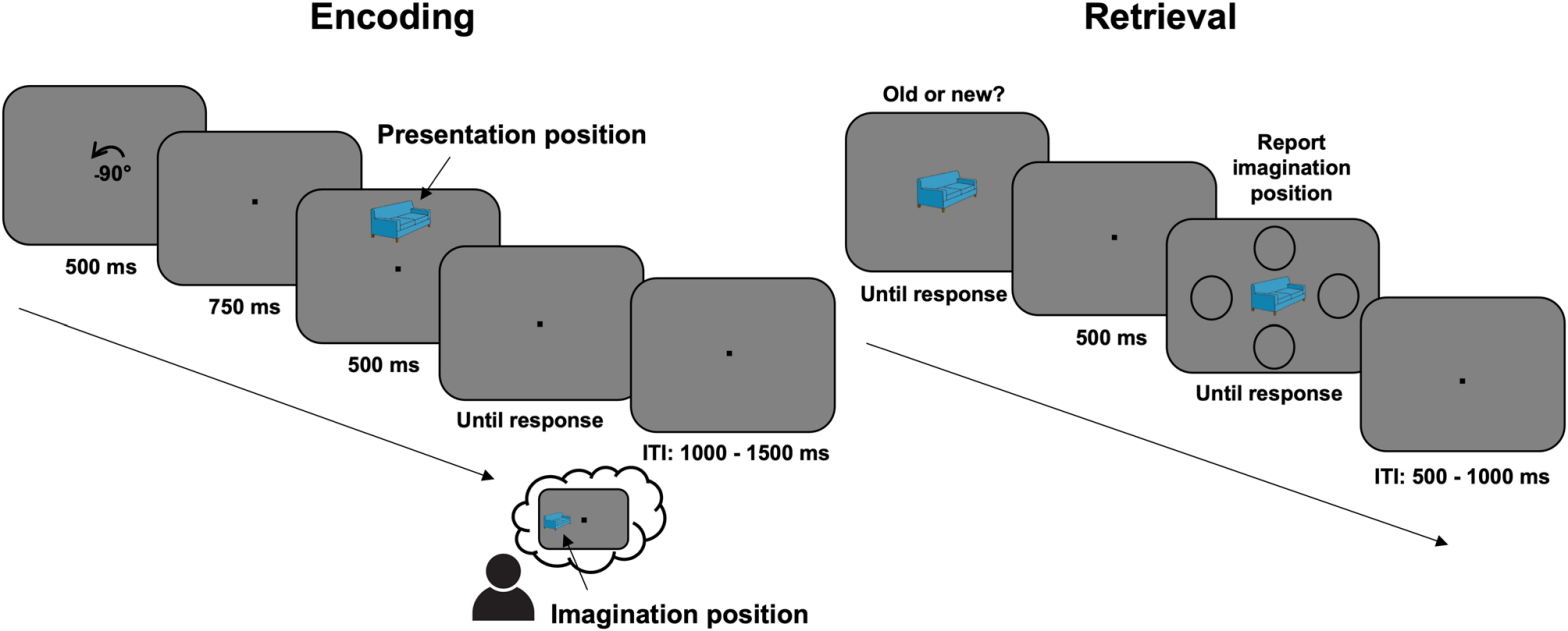
Schematic illustration of the experimental procedure. In the encoding phase, subjects were required to learn associations between everyday objects and imagination positions (top, bottom, left or right). Each object was presented three times during the encoding phase and was supposed to be associated with the same imagination position. In the retrieval phase, subjects completed first an old/new recognition task, in which objects from the previous phase were randomly interleaved with a set of 120 previously unseen objects. If participants reported being familiar with the respective item, they were asked to report the associated imagination position. At the end of each trial, an inter-trial interval (ITI) randomly varying between 1000 and 1500 ms (encoding phase) or 500–1000 ms (retrieval phase) was introduced.

The experiment consisted of three phases: an encoding phase, a distractor phase (involving counting backwards in steps of three), and finally a retrieval phase. In the encoding phase, subjects were presented with an object which could appear on four positions: left, right, top or bottom. Afterwards, the object disappeared from the screen and participants’ task was to imagine it on a new position as indicated by a cue presented prior to the object (showing + 90° or −90°). Finally, the retrieval phase started with the presentation of old or new objects appearing in the center of the screen. Participants were first required to make an old/new judgement, followed by a display indicating to report the position where the previously seen object had been imagined. The experiment was designed in a way that each trial associated two positions to the objects: a presentation position and an imagination position. The position, where the object had been presented did not contain specific task-relevant information and was thus not required for task completion. The imagination position was task-relevant, and it had to be reported during the retrieval phase. Moreover, the imagination position was always located 90° clockwise or counterclockwise compared to the original presentation position, leading to the association of a lateral (left vs. right) and a central position (top vs. bottom) to each object (Fig. 1).

**Figure 2:**
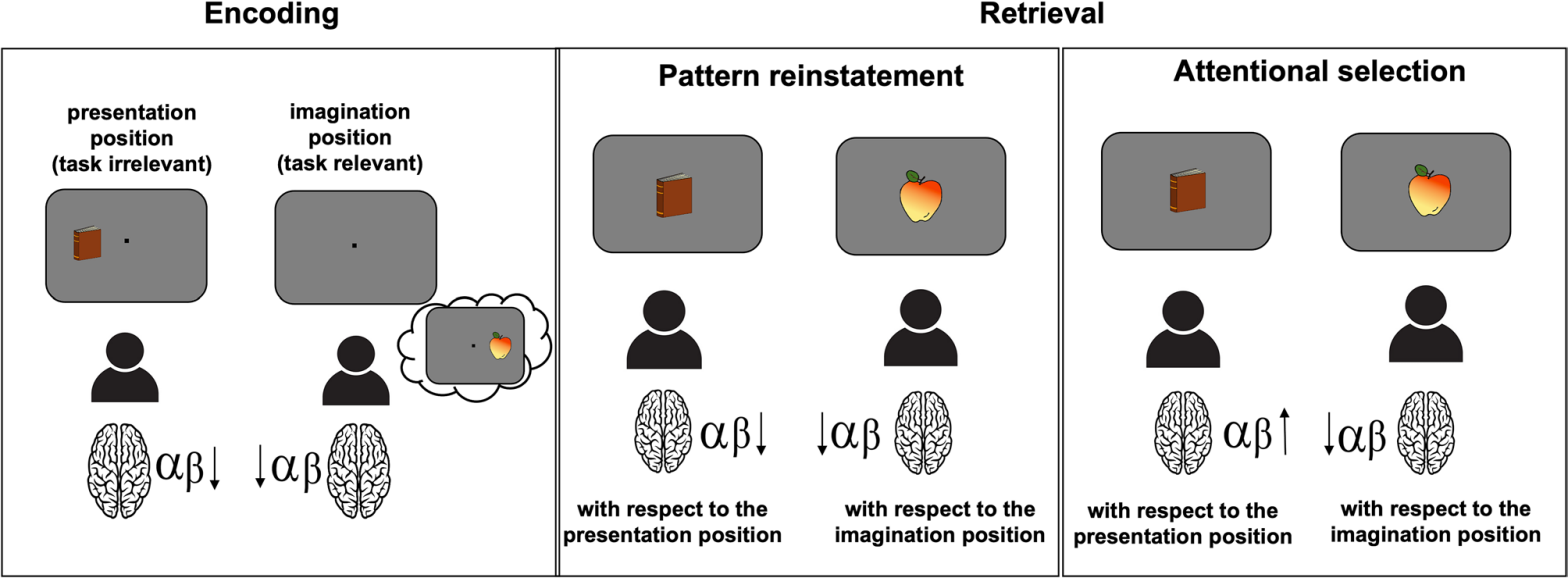
Schematic illustration of the hypotheses. Based on previous research^17,18^, we expect to find a contralateral alpha-beta decrease with respect to both the presentation position, as well as to the imagination position in the encoding phase. As for the retrieval phase, two alternative hypotheses were formulated. If lateralized alpha-beta-band activity reflects pattern reinstatement, a contralateral alpha-beta decrease is expected both with respect to the presentation and to the imagination position. However, if alpha-beta oscillations reflect attentional selection mechanisms, a contralateral alpha-beta increase with respect to the task-irrelevant information and a decrease with respect to the task-relevant information is expected.

The experiment was designed in a way that for each object the lateral position was either task-relevant (imagination position) or task-irrelevant (presentation position), which enabled us to independently measure the lateralization of alpha-beta power (8–20 Hz) in each experimental condition. If the modulation of alpha-beta oscillations as a function of the location associated with the individual object does indeed reflect spatial attentional processes, we expect the alpha-beta lateralization relative to the imagination position to differ from the lateralization relative to the presentation position. More specifically, we hypothesize a stronger contralateral alpha-beta decrease (relative to the ipsilateral hemisphere), linked to the selective retrieval of the imagination position^23–25,27^. Importantly, in line with prior research on the attentional selection of working memory content, this effect should be accompanied by a contralateral alpha-beta power increase related to the inhibition of the irrelevant presentation position^25–28^. Moreover, we assume that these effects are already evident during the retrieval of episodic information for the old vs. new decision (i.e., prior to the display requiring the imagination position report) (see Fig. 2). In addition, these alpha-beta oscillatory patterns should be accompanied by further EEG correlates previously shown to be elicited during attentional orienting. One such possibility would be the posterior contralateral negativity effect in the event-related potential (or N2 posterior contralateral; N2pc; see Luck and Hillyard^33^), which was associated with attentional selection of the task-relevant memory items^20–22^. Accordingly, we expect to observe a stronger negativity contralateral (as compared to ipsilateral) to the position subjects had to report, indicating that they directed attention to the task relevant information. Additionally, a contralateral increase in positivity (or distractor positivity; Pd; see Hickey and colleagues^34^) relative to the presentation position might indicate the inhibition of this task-irrelevant location.

However, if the modulation of alpha-beta power as a function of the location associated with the retrieved information rather reflects reinstated oscillatory patterns from the encoding phase^4^, we should be able to observe a stronger alpha-beta decrease contralateral (as compared to ipsilateral) to both the presentation and the imagination position (as both effects are expected to be present during information encoding) (see Fig. 2). Although subjects are not required to explicitly memorize the presentation position, this information is processed together with the imagination position during encoding and potentially retrieved later. Overall, the current study is suitable to provide experimental information on the extent to which oscillatory patterns measured by EEG/MEG are appropriate for representing ecphoric processes during eLTM retrieval.

## Results

### Behavioral data

**Figure 3:**
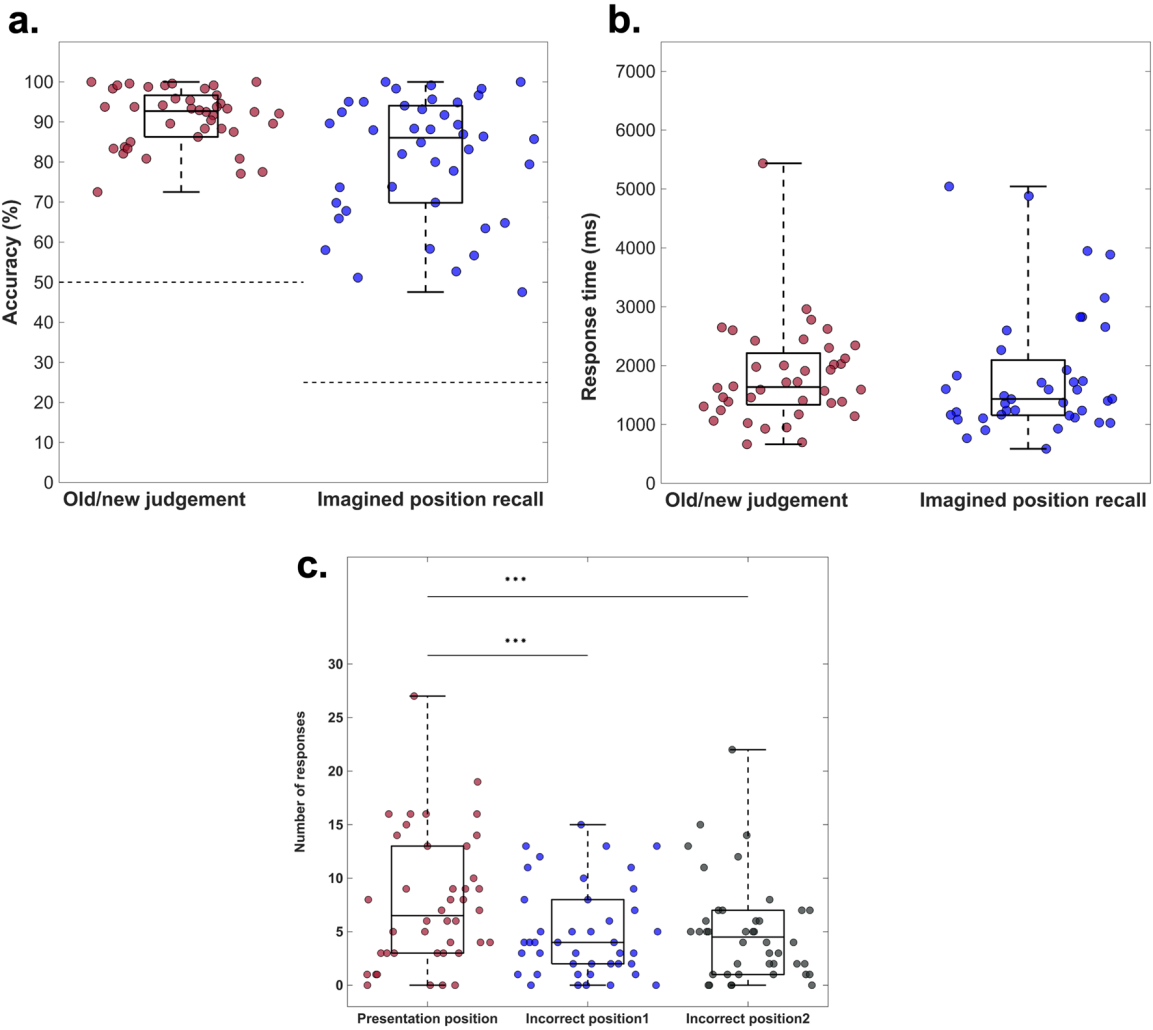
Behavioral performance. **a**. Boxplot illustrating the distribution of average accuracy (%) for the old/new recognition and imagination position recall task. All participants show above-chance performance (chance level recognition task: 50%; recall task: 25%). **b**. Boxplot showing the distribution of average reaction times (ms) for the two tasks: *M*_*recognition*_ = 1538.80 ms, *SD*_*recognition *_= 102.70 ms; *M*_*recall*_ = 1364.10 ms, *SD*_*recall*_ = 199.25 ms. **c**. Boxplot illustrating the distribution of incorrect responses. The Y-axis denotes the frequency of reporting the respective incorrect position, where the maximum value is 30.

Figure 3a depicts the accuracy distribution (in percent) for both the old/new recognition task (*M* = 91.06, *SD* = 7.12), as well as for the imagination position recall (*M* = 81.13, *SD* = 15.18). In both cases, subjects had an above chance performance: *t*(41) = 37.34, *p* < 0.001, 95% CI [88.84, 93.28] (old/new recognition task), *t*(41) = 23.96, *p* < 0.001, 95% CI [76.40, 85.86] (position recall task). Figure 3b shows the reaction time distribution for the two tasks. Moreover, we tested whether any of the incorrect positions was more frequently reported, by comparing the number of reports of each incorrect position. The repeated-measure ANOVA revealed a main effect of irrelevant position. Since sphericity was violated (χ^2^ (41) = 10.71, *p* = 0.004, ε = 0.80), Greenhouse–Geisser corrected results are reported: *F*(1, 41) = 10.33, *p* < 0.001, η_p_^2^ = 0.20. The subsequent post-hoc results revealed that participants reported the presentation position (*M* = 7.71, *SD* = 6.14) more frequently, as compared to the other two incorrect positions (*M*_*position1*_ = 4.90, *SD*_*position1*_ = 4.28; *M*_*position2*_ = 5.00, *SD*_*position2*_ = 4.74): *t*(41) = 3.81, *p*_*adj*_ = 0.001, *d*_*av*_ = 0.53, 95% CI [1.32, 4.29] (compared to incorrect position1) and *t*(41) = 3.31, *p*_*adj*_ = 0.002, *d*_*av*_ = 0.49, 95% CI [1.06, 4.36] (compared to incorrect position2). There was no difference between the frequency of reports for the remaining two incorrect positions: *t*(41) = -0.18, *p*_*adj*_ = 0.85, *d*_*av*_ = 0.02, 95% CI [-1.13, 0.94] (Fig. 3c).

### Alpha-beta power lateralization

**Figure 4:**
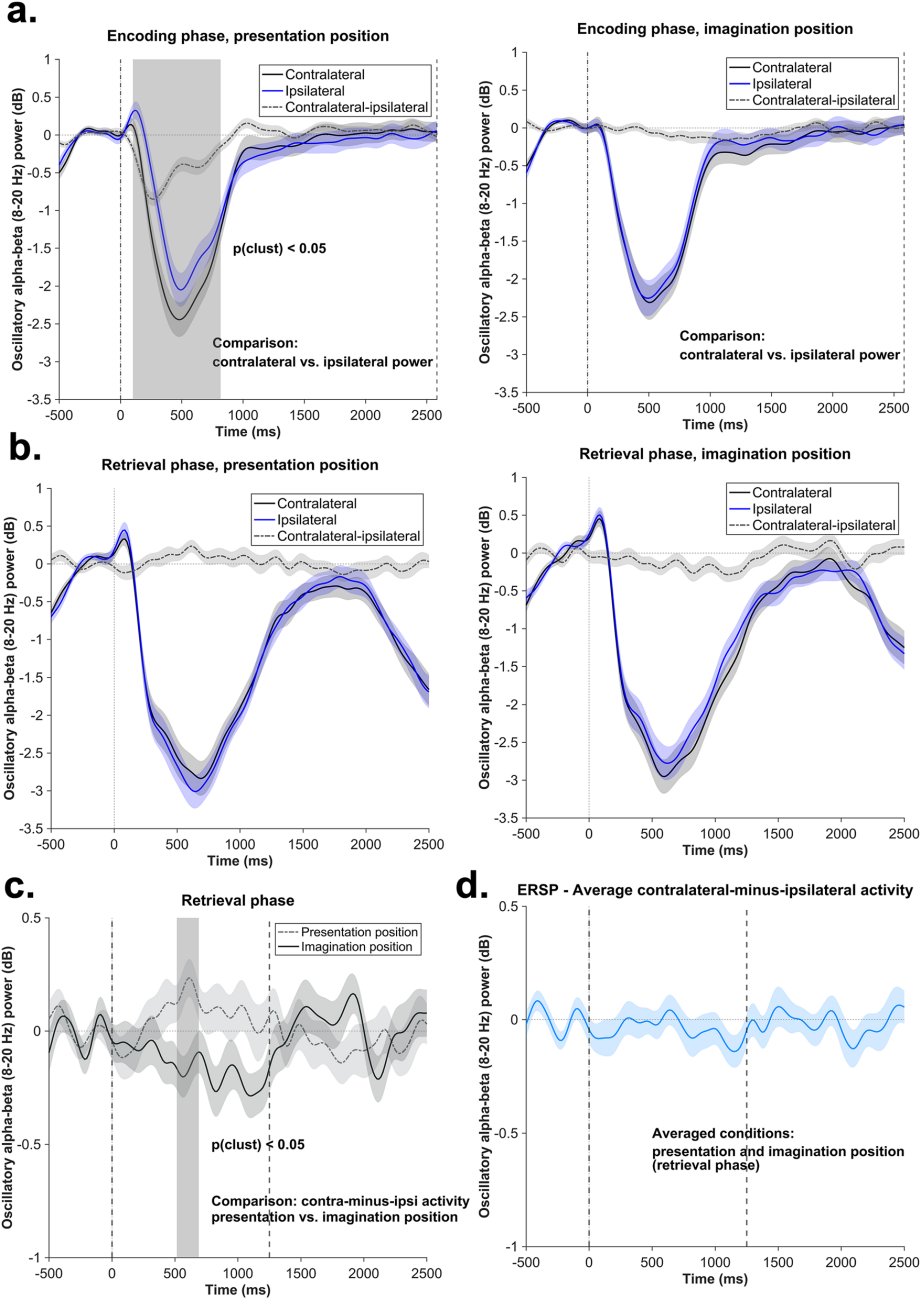
Line plot: lateralized posterior alpha-beta analysis. Data were obtained from a posterior electrode cluster: TP7/8, P5/6, P7/8, PO7/8. The shaded area represents the standard error of the mean activity. **a**. Time course of the contralateral and ipsilateral alpha-beta power (8–20 Hz) during the encoding phase, with respect to presentation position (left) and imagination postion (right). The gray area marks the significant cluster (*p* < .05) obtained as a results of the cluster-based permutation procedure used to contrast the contralateral an ipsilateral activity (time window: 0–2582 ms). **b**. Time course of the contralateral and ipsilateral alpha-beta power (8–20 Hz) during retrieval phase, with respect to the presentation position (left) and the imagination postion (right). **c**. Comparison of the contralateral-minus-ipsilateral alpha-beta power between the two conditions of the retrieval phase (i.e., relative to the presentation and imagination positions). The gray area marks the significant cluster (*p* < .05) obtained as a results of the cluster-based permutation procedure. The procedure was conducted on the 0–1250 ms time window, as indicated by the gray dotted lines. **d**. Time course of the contralateral-minus-ipsilateral power averaged for the two conditions of the retrieval phase. The cluster-based permutation procedure was conducted on the 0–1250 ms time window, as indicated by the gray dotted lines.

**Figure 5:**
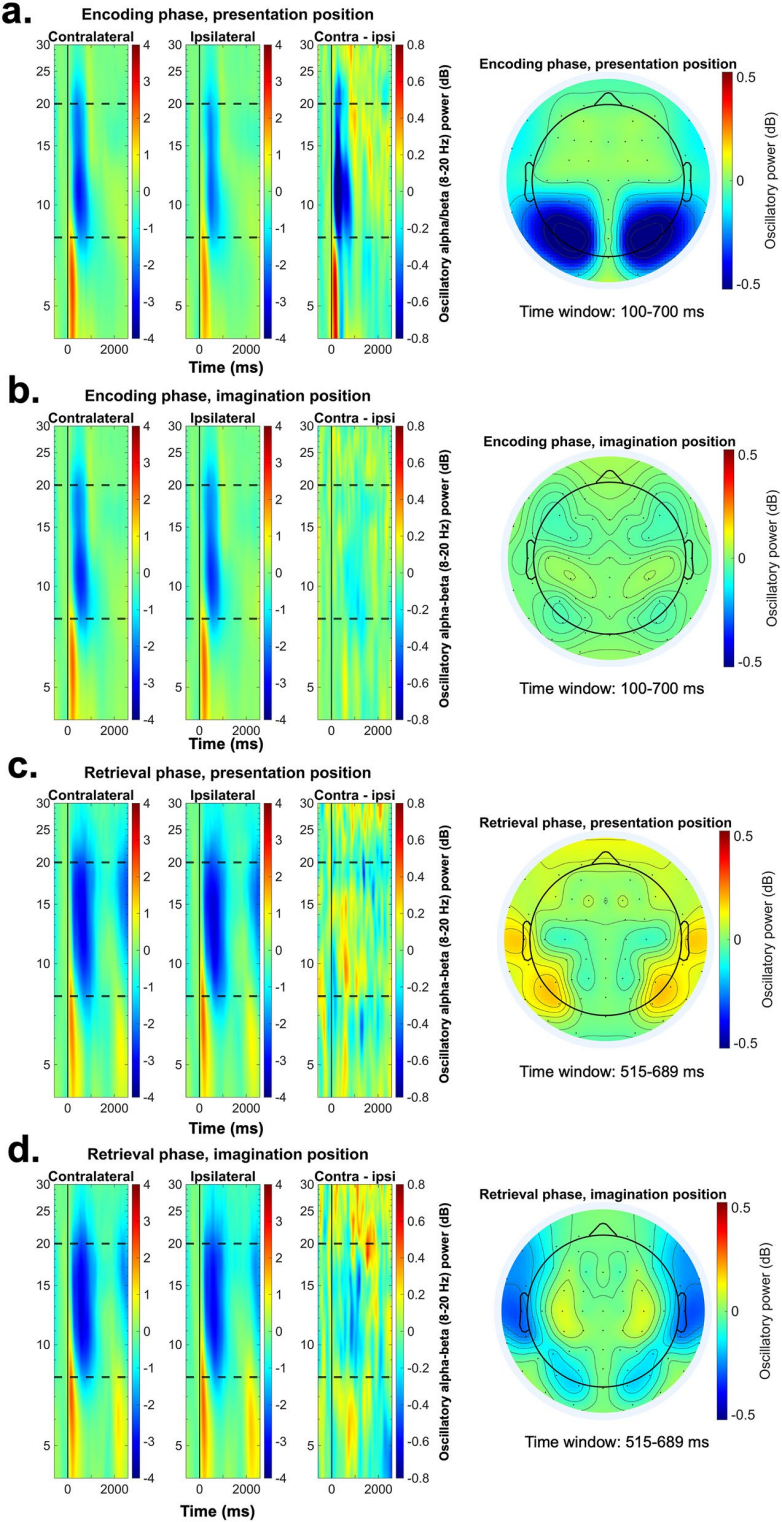
Laterlized alpha-beta analysis. **a**, **b**, **c**, **d**. The left panel depicts the time–frequency representation of the contralateral, ipsilateral, and contralateral-minus-ipsilateral data for the two phases (encoding and retrieval) and relative to the two positions (presentation and imagination). The activity-of-intereset was obtained from a posterior electrode cluster: TP7/8, P5/6, P7/8, PO7/8. Dotted lines indicate the frequencies of interest (8–20 Hz). The right panel shows the topographical distribution of the contralateral-minus-ipsilateral alpha-beta activity.

Figure 4a depicts the results of the cluster-based permutation analysis (for details see “Methods”), in which contralateral and ipsilateral alpha-beta (8–20 Hz) power was compared within each condition (i.e., relative to the presentation- and to the imagination position) in the encoding phase. Results indicated a significant cluster between 101 and 817 ms after stimulus onset with respect to the presentation position (cluster size of the original data: 46; cluster size permutation, i.e., the 95th percentile of the permuted cluster size distribution: 21), while no significant cluster (cluster size of the original data: 3; cluster size permutation: 17) was found in the comparison relative to the imagination position (see in addition Fig. 5a,b for the topographical and time–frequency representation of the encoding phase data).

For the retrieval phase analysis, two competing hypotheses were tested (see Fig. 2). To address the pattern reinstatement account, a cluster-based permutation procedure was applied to test whether the contralateral-minus-ipsilateral activity averaged across the two conditions in the retrieval phase significantly differs from zero. This analysis yielded no significant clusters (cluster size of the original data: 0; cluster size permutation: 11) (Fig. 4d). The subsequent one-sample t-test (see “Methods”) further confirms these results. As such, we found that the activity averaged over the 100–300 ms time window (*M* = −0.05 dB, *SD* = 0.43) did not differ from zero: *t*(41) = −0.82, *p* = 0.41, 95% CI [−0.19, 0.07]. Overall, this suggests that we were not able to find a statistically reliable effect to support the pattern reinstatement account.

For the second analysis (testing the attentional selection account), we contrasted the contralateral-minus-ipsilateral oscillatory power between the two conditions (i.e., the condition with a lateral imagination position was compared to the one with a lateral presentation position). The results of the cluster-based permutation analysis revealed a significant cluster between 515 and 689 ms (cluster size original data: 12, cluster size permutation: 11.5, see Fig. 4c). Importantly, in the subsequent post-hoc analyses (see “Methods”), data from this time window were averaged and tested against zero in one-tailed one-sample t-tests for each condition (see “Methods” for further details). Results showed that the contralateral-minus-ipsilateral activity significantly differed from zero in both conditions (presentation condition: *M* = 0.18 dB, *SD* = 0.45; imagination condition: *M* = −0.15 dB, *SD* = 0.53). First, we observed an alpha-beta decrease contralateral to the imagination position, suggesting that subjects selectively focused attention on the task relevant position during retrieval, *t*(41) = −1.91, *p*_*adj*_ = 0.03, 95% CI [-Inf -0.01]. Second, an alpha-beta increase was found contralateral to the presentation position, indicating that subjects inhibited the task irrelevant position, *t*(41) = 2.61, *p*_*adj*_ = 0.01, 95% CI [0.06, Inf]. These results thus indicate that lateralized alpha-beta oscillations present during eLTM retrieval reflect attentional control processes (see in addition Fig. 5c,d for the topographical and time-frequency representation of the retrieval phase data).

### Event-related potential (ERP) analysis

**Figure 6:**
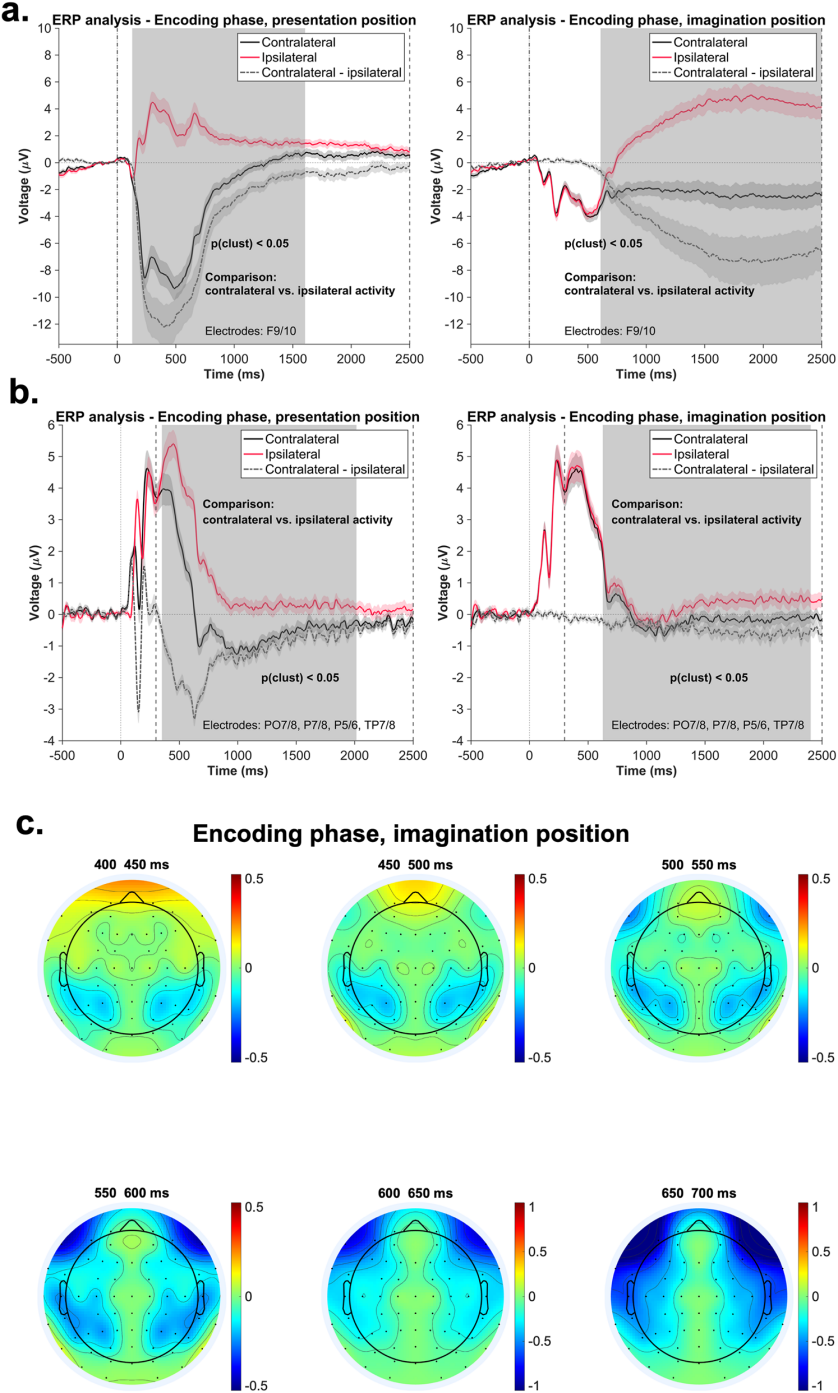
Results of the ERP analysis–encoding phase. The shaded area represents the standard error of the mean activity. **a**. Time course of the contralateral, ipsilateral, and contralateral-minus-ipsilateral frontal ERP activity during the encoding phase, with respect to presentation position (left) and imagination postion (right). The contralateral and ipsilateral activity was contrasted using a cluster-based permutation approach for the time-window: 0–2500 ms, as indicated by the dotted lines. The significant clusters are marked with gray. **b**. Time course of the contralateral, ipsilateral, and contralateral-minus-ipsilateral posterior ERP activity during the encoding phase, with respect to presentation position (left) and imagination postion (right). The contralateral and ipsilateral activity was contrasted using a cluster-based permutation approach for the time-window: 0–2500 ms, as indicated by the dotted lines. The significant clusters are marked with gray. **c**. Time course of the topographical distribution of the contralateral-minus-ipsilateral ERP activity relative to the imagination position.

In order to further explore the EEG correlates of attentional orienting during the encoding and retrieval phases of the experiment, we conducted an ERP analysis, in which we focused on both the frontal (F9/10) and posterior (PO7/8, P7/8, P5/6, TP7/8) lateralized activity. In case of the encoding phase, using a cluster-based permutation approach, we contrasted the frontal and posterior contralateral and ipsilateral activity relative to the two positions of interest. As shown in Fig. 6b, which illustrates the posterior activity, we obtained significant clusters both relative to the presentation position (351–2015 ms, cluster size of the original data: 1665; cluster size permutation: 145), and relative to the imagination position (626–2403 ms, cluster size of the original data: 592; cluster size permutation: 83.5). This increased posterior contralateral negativity shows that subjects directed attention both towards the presentation and towards the imagination positions during encoding. While there was also a strong frontal contralateral negativity (F9/10) indicative of lateral eye movements towards each of the two positions (presentation position: 128–1605 ms, cluster size of the original data: 1478, cluster size permutation: 556.5; imagination position: 609–2500 ms, cluster size of the original data: 1892; cluster size permutation: 587; Fig. 6a), the topographical plots for the imagination position (depicted in Fig. 6c) show that the frontal and posterior ERP asymmetries featured different temporal characteristics. Volume conduction from frontal to posterior recording sites can therefore be excluded as the sole cause of the posterior ERP asymmetry relative to the imagination position.

**Figure 7:**
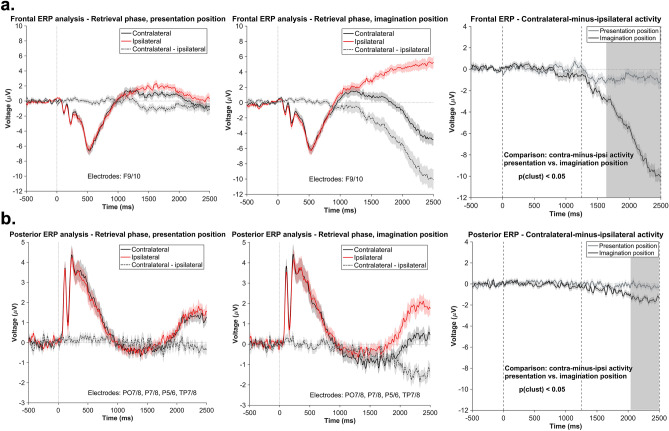
Results of the ERP analysis–retrieval phase. The shaded area represents the standard error of the mean activity. **a.** Time course of the contralateral, ipsilateral, and contralateral-minus-ipsilateral frontal ERP activity relative to the presentation position (left) and to the imagination position (middle). The right panel depicts the comparison of the frontal contralateral-minus-ipsilateral ERP activity between the two conditions. The gray area marks the significant cluster (*p* < .05) obtained as a results of the cluster-based permutation procedure. The statistical testing was conducted separately for two time windows: 0–1250 ms and 1250–2500 ms, as indicated by the gray dotted lines. **b.** Time course of the contralateral, ipsilateral, and contralateral-minus-ipsilateral posterior ERP activity relative to the presentation position (left) and to the imagination position (middle). The right panel depicts the comparison of the posterior contralateral-minus-ipsilateral ERP activity between the two conditions. The gray area marks the significant cluster (*p* < .05) obtained as a results of the cluster-based permutation procedure. The statistical testing was conducted separately for two time windows: 0–1250 ms and 1250–2500 ms, as indicated by the gray dotted lines.

In case of the retrieval phase, we contrasted the contralateral-minus-ipsilateral activity of the two conditions (relative to the presentation- and to the imagination position) for frontal (F9/10) and posterior electrodes (PO7/8, P7/8, P5/6, TP7/8) (see “Methods”). As opposed to the results from the alpha–beta power analyses, we did not find a difference in frontal or posterior ERP lateralization between the conditions with a lateral presentation vs. imagination position in the time window from 0 to 1250 ms after object presentation (frontal: cluster size of the original data: 20; cluster size permutation: 90; posterior: cluster size of the original data: 7; cluster size permutation: 57.5). However, in the later time window (1250–2500 ms), the cluster-based permutation analysis revealed a significant cluster for both the frontal lateral electrodes (1638–2500 ms, cluster size of the original data: 863; cluster size permutation: 186; see Fig. 7a right) and the posterior electrodes (2041–2500 ms; cluster size of the original data: 359; cluster size permutation: 66; see Fig. 7b right). In case of the frontal cluster, the post-hoc tests revealed that the average contralateral-minus-ipsilateral activity significantly differed from zero, both with respect to the presentation position (*M* = −0.83 µV, *SD* = 2.54), *t*(41) = −2.12, *p*_*adj*_ = 0.03, 95% CI [−1.62, −0.04], and with respect to the imagination position (*M* = −6.58 µV, *SD* = 6.08), *t*(41) = −7.01, *p*_*adj*_ < 0.001, 95% CI [−8.47, −4.68]. Regarding the posterior cluster, only the average activity relative to the imagination position (*M* = −1.44 µV, *SD* = 1.75) revealed significant results: *t*(41) = -5.33, *p*_*adj*_ < 0.001, 95% CI [−1.98, −0.89] (presentation position: *M* = −0.19 µV, *SD* = 1.38, *t*(41) = −0.88, *p*_*adj*_ = 0.37, 95% CI [−0.62, 0.24]). Summarized, while there was no evidence for ERP correlates of attentional selection or inhibition of the presentation vs. imagination position immediately after object presentation, frontal and posterior ERP lateralization later in the trial suggested an overt shift of attention (i.e., including a shift of gaze) towards these positions. The contralateral negativity in the ERP was stronger for the imagination position and thus likely reflected attentional orienting in preparation for (or during) the localization task.

### Brain-behavior correlation

**Figure 8:**
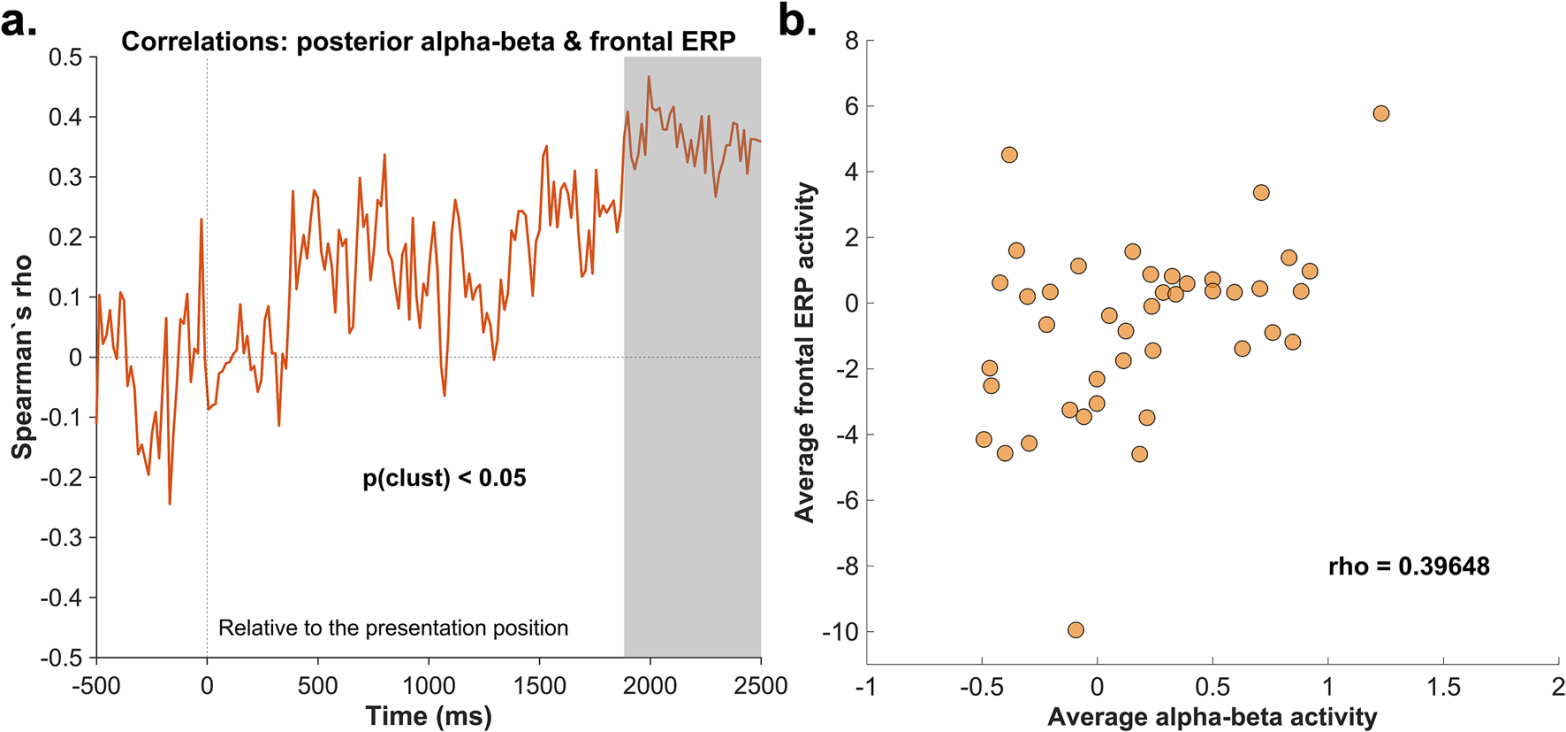
Results of brain-behavior correlations. **a.** Spearman’s correlation coefficient depicting the correlation between the average contralateral-minus-ipsilateral alpha-beta activity and frontal lateralized ERP effects (the ERP lateralization at channels F9/F10). The gray area reflects the significant cluster (*p* < .05) obtained as a result of the cluster-based permutation approach. **b.** Scatterplot of the negative correlation between the two parameters of interest averaged over the significant cluster.

The frontal ERP asymmetries in the retrieval phase suggested that participants occasionally shifted their gaze towards the relevant imagination position (and also towards the task-irrelevant presentation position). In order to assess the potential link between alpha–beta lateralization and saccadic behavior during eLTM retrieval, we conducted an exploratory correlational analysis. More specifically, we associated the average contralateral-minus-ipsilateral alpha-beta activity relative to each condition (based on the 515–689 ms cluster), with each timepoint of the frontal contralateral-minus-ipsilateral ERP activity (see “Methods” for further details). Results relative to the presentation position showed a positive correlation between the two parameters, with the cluster-based permutation procedure revealing two significant clusters in the time windows 1882–2089 ms (cluster size of the original data: 13; cluster size permutation: 13) and 2105–2502 ms (cluster size of the original data: 26; cluster size permutation: 13) (Fig. 8). This suggests that the more positive the contralateral-minus-ipsilateral oscillatory power with respect to the presentation position, the less negative the frontal ERP asymmetry. No significant results were obtained for the correlational analysis concerning the imagination position (cluster size of the original data: 13; cluster size permutation: 14).

## Discussion

The aim of the current study was to investigate whether cortical patterns present during eLTM retrieval qualify as markers of pattern reinstatement or also of attentional control. We addressed this question by measuring hemispheric asymmetries of oscillatory power in the alpha and beta frequency range and ERPs during the encoding and retrieval of objects and associated spatial positions. As such, we designed an eLTM experiment in which each object was associated with a task-relevant imagination position and a task-irrelevant presentation position, one of which was lateralized in each trial. Based on previous evidence, we formulated two alternative hypotheses. The pattern reinstatement account predicts that activity patterns during encoding and retrieval should be similar. More specifically, if lateralized alpha-beta oscillations represent a marker of pattern reinstatement, we expected to observe a stronger alpha-beta decrease contralateral (as compared to ipsilateral) to both the presentation and imagination position, as both positions had to be processed during the encoding phase. Conversely, if alpha-beta-band activity during eLTM retrieval tracks attentional control mechanisms, a stronger alpha-beta power decrease contralateral (as compared to ipsilateral) to the task-relevant imagination position and a reversed effect with respect to the presentation position (i.e., a contralaterally increased alpha-beta power) were expected during retrieval.

At a behavioral level, results indicated that subjects reported the presentation position more systematically in comparison with the other incorrect positions. This clearly supports the idea that the presentation position was encoded together with the object, despite being task-irrelevant for the imagination position recall. This is not surprising, since a considerable amount of literature argues about the special role of spatial location in organizing information in memory^35–37^. In line with this, prior research suggested that even spatial information associated with only passively viewed stimuli is encoded into eLTM and can be retrieved in a later recall task^4^. The phenomenon we observed at the behavioral level is analogous to that of committing swap errors in working memory paradigms (i.e., erroneous report of task-irrelevant working memory content)^38^. It has been suggested that these swap errors occur with higher frequency as the memory load increases^39,40^ and are possibly due to the confusion of the target feature with highly similar non-target information^41^. In the context of the current experiment, it seems possible that the remaining residual information of the task-irrelevant presentation position interfered with the target report, thus leading to a more systematic bias towards the presentation position, especially in cases of poor memories.

The lateralized alpha-beta analysis conducted for the encoding phase revealed a stronger contralateral decrease with respect to the presentation position (as compared to the ipsilateral oscillatory power), suggesting that subjects moved the focus of attention to the object’s position^15–17^. This was expected, since the visual objects were presented unilaterally (i.e., they drew attention in a bottom-up way^4^). A slight alpha-beta power asymmetry was also found with respect to the imagination position, but it did not reach significance in the current sample. Lateralized alpha-beta activity was also observed during the retrieval phase. The contralateral-minus-ipsilateral power differed between the presentation and imagination conditions, showing a contralateral-minus-ipsilateral suppression relative to the task-relevant imagination position and a reversed effect relative to the task-irrelevant presentation position. This suggests that the two lateralized effects are linked to separate mechanisms, both facilitating eLTM retrieval. According to prior research on the attentional selection of working memory contents^23,25,27^, this pattern of results can be considered as evidence for attentional re-focusing on the task relevant imagination position (reflected by the contralateral alpha-beta power decrease) and the inhibition of the task-irrelevant presentation position (reflected by the contralateral alpha-beta power increase). As indicated by the behavioral results, it seems plausible that the presentation position was also encoded together with the imagination position into eLTM, despite being task irrelevant. As such, the centrally presented objects in the retrieval phase (acting as a retrieval cue) triggered the reactivation of the associated imagination position, which was accompanied in certain trials by an automatic reactivation of the presentation position, a process presumably interfering with the retrieval of the task-relevant spatial information. In this context, it seems plausible that the interference was reduced by attentional control processes, required for inhibiting irrelevant information and for selecting the relevant spatial position for the later report.

Further evidence in support of this interpretation was provided by the lateralized ERP activity patterns. For the encoding phase, lateralized ERP patterns at frontal electrodes indicated strong saccades when processing the unilaterally presented items and later when imagining it on a new position. Importantly, the posterior contralateral negativity indicated that subjects also covertly directed attention towards the imagination position (see Fig. 6), although no reliable effect has been found in the alpha-beta lateralization in this regard. This ERP effect is in line with previous literature, which linked posterior contralateral negativity to attentional orienting within memory representations^20–22^. In the context of the current task, subjects were specifically instructed to fixate on the centrally presented point; however it seems that their gaze was still shifted towards the attended locations. This phenomenon is consistent with findings indicating that saccades and microsaccades (i.e., small saccades happening during fixation) occur during attentional orienting to perceptual stimuli^42^, as well as during attentional selection of relevant memory representations^43,44^.

The ERP results for the retrieval phase revealed that subjects overtly directed attention towards the imagination position and also, but to a lesser extent, towards the presentation position. These effects appeared in rather late time windows (relative to object presentation), suggesting an attentional process linked to the object localization task. To test whether the alpha-beta asymmetry following object presentation was related to these later lateralized effects in the ERP, we conducted an additional exploratory correlational analysis. For the presentation position, our results showed a significant cluster of positive correlations only in a later time window of the trial, suggesting that subjects who exhibited a stronger inhibitory alpha-beta effect, had a lower tendency to later make saccades towards the presentation position. Since the alpha-beta asymmetry preceded the lateralized ERP activity at frontal sensors, it seems plausible that a more efficient inhibitory process might have led to a weaker representation of the task-irrelevant position during the imagination position report.

The pattern of results obtained in the current study corroborate the findings provided by experiments investigating attentional control on the level of working memory. It was argued that attentional control is involved in monitoring the attentional switches between memory representations, a process which is tracked by lateralized alpha activity. More specifically, task-relevant items entering the focus of attention were shown to elicit a stronger contralateral alpha suppression, compared to the ipsilateral hemisphere, while the currently irrelevant items are related to a stronger contralateral alpha increase^25,26,45^. Considering the similarity of these findings with the current result patterns, it seems plausible that upon object presentation, subjects retrieved both spatial associations (i.e., presentation and imagination positions) from long-term memory. Subsequently, in support for the position report, this information was brought back into working memory, where the task-relevant position was selected, while the task-irrelevant one was inhibited. In this context, it seems likely that the attentional selection mechanisms acting at the level of working memory facilitated the process of long-term memory retrieval.

This interpretation is also in line with an influential model of memory (i.e., Embedded process model of working memory), which states that successful information retrieval from eLTM is realized by passing the respective information through the focus of attention in working memory^46^. In accordance with this theory, Fukuda and colleagues^47^ provided experimental evidence suggesting that information retrieved from long-term memory is brought back into working memory, where it is temporarily stored. As such, the authors discuss about the existence of two different inter-connected memory states relevant for the retrieval process: an active state of information representation in working memory and a passive state of information storage in long-term memory. Importantly, a recent study conducted by Vo and colleagues^48^ further supports the idea of working memory and long-term memory retrieval being connected. In their study, authors demonstrated that activity in the occipital (V1–V4 areas) and parietal cortex (V3AB, IPS0-2 retinotopic regions) during working memory maintenance and long-term memory retrieval is highly similar^48^. Moreover, this conclusion is fully in line with the results of an earlier study, in which a classifier trained on the cortical activity recorded during a long-term memory task performed above chance when tested on the delay period of a working memory task^49^. Authors interpreted their results as evidence for the long-term memory system supporting information maintenance in working memory.

The present results have important implications for the study of eLTM retrieval. First, we provide experimental evidence supporting the active role of selective attention during eLTM retrieval, a process, which can be easily confounded with pattern reinstatement, especially when assessing neural correlates of retrieval based on cortical activity. As such, we question whether brain modulations recorded on the scalp surface by EEG/MEG techniques constitute a suitable method for the investigation of pattern reinstatement. Since previous evidence shows that hippocampal pattern completion and MTL activity mediate the process of pattern reinstatement^11,50^, it seems plausible that the hippocampus and some parts of the MTL are necessary for cortical reinstatement. Therefore, brain imaging techniques such as intracranial EEG or fMRI (which can record activity patterns in the MTL or the hippocampus with higher precision), are more suitable to study pattern reinstatement. Future studies aiming to investigate this phenomenon during eLTM should carefully consider two important aspects: (i) the choice of the brain imaging technique; and (ii) the construction of experiments, which allow for an adequate control of overlapping processes, such as selective attention.

### Conclusions

To summarize, the current study aimed to shed light on the functional role of analogous activity patterns during eLTM encoding and retrieval. Namely, we addressed the question of whether these modulations reflect pattern reinstatement or attentional control mechanisms. Our results supported the latter account, showing that subjects selected the task relevant information, while they inhibited the task irrelevant presentation position during eLTM retrieval. Since our results regarding the pattern reinstatement account were not conclusive, we cannot exclude the possible contribution of this phenomenon to the scalp distributions of alpha-beta oscillations during eLTM retrieval. Overall, these results raise an important issue for the study of eLTM retrieval: there is a need to differentiate and control for the effects of attentional selection processes when investigating the neural correlates of pattern reinstatement.

## Methods

### Participants

Originally, data from 46 participants was collected. Four participants had to be excluded from the final analysis: three of them were not able to complete the task using the given visual strategy and in the case of the fourth one, technical issues with the EEG system were encountered. The remaining 42 participants (22 female, 20 male) had an age between 18 and 30 years (*M* = 24.1 years, *SD* = 2.83), were right-handed, had normal or corrected-to-normal vision, and did not suffer from any neurological or psychiatric disorders. For their participation, subjects received a compensation either as a payment of 10 €/hour, or study participation credits (required for the completion of a bachelor’s degree in psychology). Prior to participation, written consent was obtained from all subjects. The study was in accordance with the Declaration of Helsinki and had been approved by the ethics committee of the Leibniz Research Centre for Working Environment and Human Factors (Dortmund, Germany).

### Procedure

Upon arrival, participants were presented with general information about the study, followed by the completion of the German version of the following questionnaires: a demographic survey and the Edinburgh Handedness Inventory^51^. As a next step, the EEG cap was set and subjects were led into the sound-attenuated, electrically shielded, and dimly lit EEG laboratory.

The experiment was performed on a 22-inch CRT monitor (100 Hz; 1024 × 768 pixels), with a viewing distance of approximately 145 cm. The task was realized in Lazarus IDE (Free Pascal) and presented using ViSaGe MKII Stimulus Generator (Cambridge Research Systems, Rochester, UK). The eLTM task included a short training and three phases: encoding, distractor, and retrieval phase. Once the task was finalized, subjects completed a follow-up questionnaire, in which they were asked about the strategy they used and difficulties they encountered while completing the experiment. The whole session lasted between 3 and 3.5 h.

### Stimulus material

A total number of 240 everyday objects were used for the eLTM task^52^. Since the original object pool consisted of 260 images, a prior image selection process was conducted. First, images depicting objects pointing towards certain directions (e.g., a gun, a pointing finger) were excluded (seven objects in total). Secondly, we conducted a survey, in which an independent sample of subjects rated the remaining 253 objects based on a set of criteria (see details in the next section). Based on these results, 13 more objects were removed from the final pool of images.

#### Image rating survey

The image rating survey contained three statements, each reflecting the agreement of subjects with respect to: (i) proper luminance, contrast and vividness of the object; (ii) ease of recognizability; (iii) complexity. Subjects’ task was to rate all objects based on these three statements. Answers were given on a 5-point Likert scale (for statements one and two: 1 = “Total disagreement”, 5 = “Total agreement”; for statement three: 1 = “Simple”, 5 = “Complex”). The aim of the image rating survey was to identify the objects, which stood out in terms of sensory quality (reflected by luminance, contrast, and vividness) and those, which were easily recognizable. In addition, we aimed to obtain measures of complexity, which were later used to counterbalance the stimuli in the eLTM task.

A total number of 30 participants completed the questionnaire (18 females, 10 males, and 2 unspecified gender). Their age ranged between 18 and 35 years (*M* = 27 years, *SD* = 4.38). The survey was realized in PsychoPy3^53^ and uploaded online to Pavlovia (https://pavlovia.org/). The questionnaire was completed by volunteers, who did not receive compensation for their participation.

In order to identify the 13 to-be-removed objects, a score reflecting the mean object quality (statement one) and recognizability (statement two) was calculated (*M* = 4.57, *SD* = 0.25). The objects obtaining the 13 lowest scores (smaller than 4.07) were excluded from the stimulus pool. Furthermore, for categorizing objects based on complexity, we conducted a median split analysis on the remaining 240 images, resulting in a pool of 120 simple and 120 complex objects.

### Episodic long-term memory task

The experimental task started with a training, which contained 27 encoding trials and 19 retrieval trials and aimed to familiarize subjects with the experimental procedure. Thus, data acquired during these practice trials was excluded from the final analysis. Once the training was complete, subjects performed the encoding phase. Each encoding trial started with a cue, presented on a gray background (RGB: 128, 128, 128) depicting either + 90° (i.e., the imagination position is situated clockwise relative to the presentation position) or −90° (i.e., the imagination position is situated counterclockwise relative to the presentation position). Following a 750 ms inter-stimulus interval (ISI), objects (size: 4.3° × 3°) were presented on one of the four positions on the screen (top, bottom, left, right). The distance between the object and the central fixation point was 3° visual angle. After 500 ms, the object disappeared and the subject’s task was to imagine the object on a new position, as indicated by the cue shown at the beginning of the trial. For instance, if the cue showed + 90° and the object was presented at the top position, participants had to imagine the object on the right position. Both during object presentation and the imagination task, participants were instructed to fixate on the centrally presented black dot (size: 0.2° × 0.2°). The imagination task was self-paced. After each trial subjects had to confirm through a mouse click that they finished imagining the object on the corresponding position on the screen. The inter-trial interval (ITI) varied randomly between 1000 and 1500 ms (Fig. 1).

The encoding phase consisted of 360 trials, organized in six blocks (60 trials/block). Between blocks, self-paced breaks were introduced. Each participant learnt 120 object-imagination position associations. In order to facilitate the learning process, each object occurred in three trials, each time on the same position and having the same imagination position associated with. However, trials showing the same objects could not be in the same block. The object-imagination position associations were randomized across subjects, while object complexity and format (landscape vs. portrait) were counterbalanced across the imagination condition.

The encoding phase was followed by a distractor phase, which required participants to count backwards starting from 500, in steps of three. The distractor phase had a duration of three minutes and was preceded and followed by three-minute breaks. Finally, in the retrieval phase, subjects were shown a centrally presented object (0.2° × 0.2°) and their task was to decide whether they had already seen the respective item (old/new judgement). Half of the items presented in this phase were also shown in the encoding phase, whereas the rest of them were unknown to participants. Responses were given with the left and right button of the computer mouse each button being associated with a response type (old or new). The mapping between the button and the response was counterbalanced across subjects. In trials, in which subjects indicated familiarity with the object, the associated imagination position had to be further reported by clicking on a circle (diameter: 2°) associated with the remembered position. The distance between the central object and each of the four circles was 3° visual angle. Trials in the retrieval phase were separated by an ITI of 500–1000 ms. The retrieval phase consisted of 240 trials in total (120 old and 120 new items), organized in 4 blocks which were separated by self-paced breaks (Fig. 1).

### Data analysis

All analyses were conducted in MATLAB® (R2019b).

#### Behavioral analysis

As a first step in the behavioral analysis, we tested whether participants’ performance (as reflected by the average accuracy) in the old/new recognition and the imagination position recall task were above chance. In both cases, we conducted a separate one-sample t-test, in which we contrasted the average accuracy to 50% (old/new judgement) and 25% (imagination position recall). Furthermore, in order to investigate whether subjects reported more frequently any of the incorrect positions, the number of reports corresponding to each incorrect position was compared using a one-way analysis of variance (ANOVA). Only trials, in which subjects provided both a familiarity judgement (i.e., reported whether the object was old or new) and a valid imagination position report (i.e., clicked within the circle representing the imagination position) were included in the behavioral analysis. Failure of clicking within the indicated circle led to trials with missing responses, which were excluded from further analysis.

#### EEG recording and preprocessing

EEG data were recorded using a 64 Ag/AgCl passive electrode system (Easycap GmbH, Herrsching, Germany). The electrodes were distributed according to the extended 10/20 System^54^ and the signal was amplified by a NeuroOne Tesla AC-amplifier (Bittium Biosignals Ltd, Kuopio, Finland). Data were sampled at a rate of 1000 Hz and a 250 Hz low-pass filter was applied during recording. AFz served as ground electrode, FCz as reference electrode. Throughout the recording session, impedances were kept below 20 kΩ. For the EEG data analysis, we used the EEGLAB toolbox^55^ (v. 14.1.2) implemented in MATLAB® (R2019b). Before preprocessing, each subject’s dataset was separated into an encoding and a retrieval dataset, so subsequent analyses were done separately on the encoding and retrieval data. Moreover, in order to speed up the analysis of the retrieval dataset, we only included trials showing objects present during the encoding phase (i.e., old objects).

As a first preprocessing step, data was 0.5 Hz high-pass (filter order: 6600, transition band-width: 0.5 Hz, cutoff frequency at −6 dB: 0.25 Hz) and 30 Hz low-pass filtered (filter order: 440, transition band-width: 7.5 Hz, cutoff frequency at −6 dB: 33.75 Hz) using a Hamming windowed sinc FIR filter (i.e., pop_eegfiltnew from the EEGLAB toolbox). Next, using the automated channel rejection procedure implemented in the toolbox (i.e., pop_rejchan), artifact channels were rejected (range: 0–6, Encoding dataset: *M* = 2.33 channels, Retrieval dataset: *M* = 2.04 channels), with the use of the following parameters: kurtosis of each channel as a rejection measure, outlier selection based on 15 standard deviations. Next, the data were re-referenced to the average activity of the remaining electrodes.

In order to prepare the data for the independent component analysis (ICA), the data were further downsampled to 200 Hz, 1 Hz low-pass filtered (filter order: 660, transition band-width: 1 Hz, cutoff frequency at −6 dB: 0.50 Hz) using a Hamming windowed sinc FIR filter and epoched. For both the encoding and the retrieval dataset, the chosen time window was −1000 to 3000 ms, time-locked to the object’s onset. Afterwards, the data were baselined to the 200 ms time window prior to stimulus onset and an automated trial rejection procedure implemented in EEGLAB (i.e., pop_autorej; threshold: 500 μV, maximum % of rejected trials: 5%) was used to reject trials containing extreme fluctuations (encoding phase: *M* = 50.04 trials, 15.35%; retrieval phase: *M* = 8.16 trials, 10.85%).

Following, ICA was conducted on the rank reduced data (remaining number of channels minus one). Dimensionality reduction was achieved using the principal component analysis implemented in EEGLAB. Next, the obtained IC weights were transferred to the bandpass-filtered, average re-referenced data. In order to identify independent components (IC) reflecting blinks, horizontal- and vertical eye movements, and general discontinuities, we used the ADJUST plugin^56^ (version 1.1.1). In addition, in order to identify the ICs containing high residual variance, we estimated a single-equivalent current dipole model, using the DIPFIT plug-in. Thus, further components exceeding 40% residual variance regarding their dipole solution were excluded. Overall, 15.95 IC components were marked for exclusion in the encoding (range: 6–35) and 15.42 in the retrieval dataset (range: 3–25). Once data was epoched and baseline-corrected (same parameters as above), the marked IC components were removed. Afterwards, trials left with extreme fluctuations were excluded from the analysis using the automated approach mentioned above (threshold: 1000 μV, maximum % of rejected trials: 5%): on average 74.40 trials (20.50%) were excluded from the encoding dataset and 12.90 trials (14.26%) from the retrieval dataset. Finally, channels excluded at the beginning of the analysis were interpolated using a spherical spline interpolation technique.

For the subsequent EEG analyses, we included all trials of the encoding and retrieval phase which passed the trial rejection procedure during preprocessing. In case of the retrieval phase, two additional criteria were considered for trial inclusion: (i) correct identification of the old object; (ii) providing an old-new decision within 3000 ms.

#### Time-frequency decomposition

Time–frequency decomposition was done by convolving each trial of the EEG data with 3-cycle complex Morlet wavelets. A complex Morlet wavelet is defined as a complex sine wave that is tapered by a Gaussian. The obtained epochs contained 200 time points in the time window −582 to 2582 ms relative to the object onset (for both leaning and retrieval phase) and the frequencies ranged between 4 and 30 Hz, increasing in logarithmic steps of 26. The number of cycles, which defines the width of the tapering Gaussian, increased linearly as a function of frequency by a factor of 0.5. As such, the number of cycles for the lowest frequency (4 Hz) was three, whereas 11.25 cycles were used for the highest frequency (30 Hz). A time window between -500–0 ms was chosen as a spectral baseline. The time-frequency decomposition was conducted through the STUDY functions of EEGLAB (i.e., std_precomp). For this analysis, all encoding phase trials remaining after the trial rejection procedure (conducted during preprocessing) were included in the STUDY. In case of the retrieval phase data, we additionally excluded the trials, in which subjects failed to correctly identify the old objects. Moreover, trials in which subjects’ reaction time in the recognition task was greater than 3 s were also excluded.

#### Alpha-beta power lateralization

In order to answer our main research question, lateralized modulations of posterior alpha-beta (8–20 Hz) power were assessed^4^. As a first step in our analysis, we focused on the activity patterns of the encoding phase. This activity was obtained by averaging the power values across the frequency band of interest (8–20 Hz) and across a lateral posterior electrode cluster, consisting of PO7/8, P7/8, P5/6, TP7/8. The electrode selection procedure was based on previous findings on attentional selection within working memory^20,27,57–59^. The mean contralateral and ipsilateral power was separately calculated with respect to the presentation and imagination position. To assess the difference between the contralateral and ipsilateral activity within each condition (i.e., relative to the presentation position and to the imagination position), we applied a cluster-based permutation approach for each condition separately. While we expected that a stronger contralateral (compared to ipsilateral) suppression of alpha-beta power would be present in different time windows for the presentation position and imagination position, we were uncertain how late such an effect would occur for the latter condition. The procedure was thus conducted on the time window 0–2582 ms. First, the power value of the contralateral and ipsilateral activity was compared at each of the 163 time points using paired-sample t-tests. This resulted in a vector of 163 *p*-values corresponding to each comparison of the original data. Based on this vector, clusters containing more than one significant *p*-value (i.e., lower than 0.05) were obtained. In the following step, we generated a distribution of maximum cluster sizes. This was achieved by randomly re-shuffling the condition labels (i.e., contralateral and ipsilateral) 1000 times and in each iteration paired-sample t-tests were used to compare the ipsilateral and contralateral activity at all time points. Based on the vector of 163 *p*-values, the cluster containing the largest number of significant *p*-values (i.e., smaller than 0.05) was subtracted and saved for each iteration, thus resulting in a distribution of maximum cluster sizes. Finally, we determined the 95th percentile of the cluster size distribution and clusters bigger than this cutoff value were considered significant.

In order to address the two competing hypotheses regarding the alpha-beta oscillatory patterns during the retrieval phase (see Fig. 2), two separate analyses were conducted. First, the pattern reinstatement account predicts a contralateral alpha-beta decrease with respect to both the presentation and imagination positions (see Fig. 2 middle). To test this assumption, we calculated the mean contralateral and ipsilateral activity relative to the initial presentation- and imagination position. Similar to the encoding phase analysis, data were obtained from the lateral posterior electrode cluster (PO7/8, P7/8, P5/6, TP7/8), by averaging the activity across the 8–20 Hz frequency band. Afterwards, we calculated the contralateral-minus-ipsilateral activity and averaged it across the two relevant conditions (i.e., with respect to the presentation- and imagination position). Subsequently, the average power values were contrasted to zero using the same cluster-based permutation procedure described above (time window: 0–1250 ms). Then, using a one-sample t-test, we assessed whether the same activity averaged over the time window 100–300 ms significantly differs from zero. The choice of the time window was motivated by previous EEG/MEG studies, which showed that cortical patterns from the encoding phase are reinstated during memory retrieval ~ 100–300 ms after object presentation^4,7,60^.

Second, we tested the attentional selection account, according to which we expected different patterns in posterior alpha-beta lateralization for the presentation position and the imagination position: (i) a stronger contralateral (as compared to ipsilateral) alpha-beta power decrease relative to the imagination position; and (ii) a stronger contralateral (as compared to ipsilateral) power increase relative to the presentation position (see Fig. 2 right). To determine a time window during which these lateralized effects could be tested, the contralateral-minus-ipsilateral difference waves obtained with respect to the imagination and presentation position were contrasted using the same cluster-based permutation approach (see above). This procedure was restricted to the time window from 0 to 1250 ms (after object presentation). Although previous research studying EEG correlates of selective attention within working memory showed that the respective effect extends until about 800 ms after stimulus presentation^61,62^, a longer time window was chosen in the context of the current long-term memory paradigm. In case of a significant effect, we conducted *t*-tests against zero based on the activity averaged over the significant cluster, with the aim of testing whether a contralateral suppression (imagination position) or a contralateral increase in alpha-beta power (presentation position) was evident.

#### ERP analyses

In addition to the lateralized effects in the alpha-beta frequency domain, we also looked at lateralized effects in the event-related potentials (ERPs). For the purpose of this analysis, the ICs assigned to lateral eye movements by the ADJUST procedure^56^ were not excluded. The rest of the preprocessing steps matched the steps already described in the Methods section.

For the encoding phase, we analyzed both the posterior and the frontal lateralization in the ERP activity. While the posterior effects were studied as correlates of attentional selection processes^20–22^, frontal asymmetries were used as a correlate of lateral eye movements (saccades). As a first step, we calculated the mean contralateral and ipsilateral activity relative to the initial presentation and imagination position. For the posterior ERP analysis, data were averaged across the same lateral posterior electrode cluster used for the alpha–beta analysis (i.e., PO7/8, P7/8, P5/6, TP7/8). For the frontal ERPs, data from channels F9/10 were used. In order to contrast the posterior contralateral and ipsilateral activity in each of the two conditions (i.e., with respect to the presentation and imagination positions), the same cluster-based permutation procedure described above was applied. In case of the frontal electrode cluster, the analysis was conducted on the time window 0–2500 ms, while in the case of the posterior cluster, the analysis restricted to 300–2500 ms. The choice of 300 ms as the lower limit was motivated by the presence of early posterior asymmetries, which were due to the lateralized presentation of the visual objects. These early ERP effects were of no interest for the current investigation.

With respect to the retrieval phase, analogous posterior (PO7/8, P7/8, P5/6, TP7/8) and frontal ERP lateralization (F9/10) were calculated. Comparable to the alpha-beta analysis, the attentional selection account would predict different patterns of lateralized ERP effects relative to the presentation position and relative to the imagination position following object presentation. To test for this, we contrasted the contralateral-minus-ipsilateral activity with respect to the two positions, separately for the frontal and posterior clusters. Comparable to the alpha-beta analyses, this was done within a time window from 0 to 1250 ms after object presentation. We additionally used a later time window from 1250 to 2500 ms to test whether any covert shifts of attention or eye movements occurred in preparation for (or during) the imagination position report. Finally, using one-sample t-tests, we conducted a post-hoc analysis in which we compared the condition-wise averaged activity from the significant clusters to zero. It must be noted that in all the above-mentioned statistical procedures the significance level was set to *p* < 0.05.

#### Brain-behavior correlations

A further exploratory correlational analysis was conducted in order to investigate the potential influence of the alpha-beta activity on saccadic behavior (as reflected by frontal ERP asymmetries) during retrieval. More specifically, the alpha-beta parameter was obtained by averaging the contralateral-minus-ipsilateral activity of the significant time window (515–689 ms) resulting from the cluster-based permutation analysis (see Fig. 4c). Following, Spearman correlations were calculated between the alpha-beta parameter and each timepoint of the frontal contralateral-minus-ipsilateral ERP activity. This procedure was run separately for the ERPs and alpha-beta power asymmetries relative to the imagination and presentation position. In order to correct for multiple comparisons, we applied a very similar cluster-based permutation analysis to the one described above. However, in the current procedure, instead of re-shuffling the condition labels, we re-shuffled the ERP values corresponding to each participant. The procedure was conducted on the entire time window (0–2500 ms), with a significance level set to *p* < 0.05.

#### Inferential statistics and effect sizes

All statistical tests and measures of effect size were obtained in MATLAB® (R2019b). For the one-way ANOVA comparing the frequency of report for each incorrect position, Mauchly’s test for sphericity was applied. Because the sphericity assumption was violated, the Greenhouse–Geisser correction was introduced (denoted with ε). As a measure of effect size, we calculated partial eta squared (η_p_^2^). Regarding the applied t-tests, two-tailed *p*-values were calculated, if not otherwise specified. As a measure of effect size, we followed the recommendations of Lakens^63^ and computed Cohen’s *d*_*av*_. In the case of post-hoc tests requiring a correction for multiple comparison, false discovery rate (FDR) corrected *p*-values were provided (denoted by *p*_*adj*_*)* ^64^.

## Supplementary Information

Supplementary Information 1.

Supplementary Information 2.

Supplementary Information 3.

## Data Availability

The data and all the scripts used for the reported analyses are publicly available on Open Science Framework (OSF) accessing the following link: https://osf.io/ngdzm/?view_only=018451896c84482e8df189cee11646a8.
