# Supplementary figures and images for: Pattern reinstatement and attentional control overlap during episodic long-term memory retrieval
Source: Sci Rep. 2022 Jun 24;12:10739. doi: 10.1038/s41598-022-14090-4 (PMC9232640; doi:10.1038/s41598-022-14090-4)

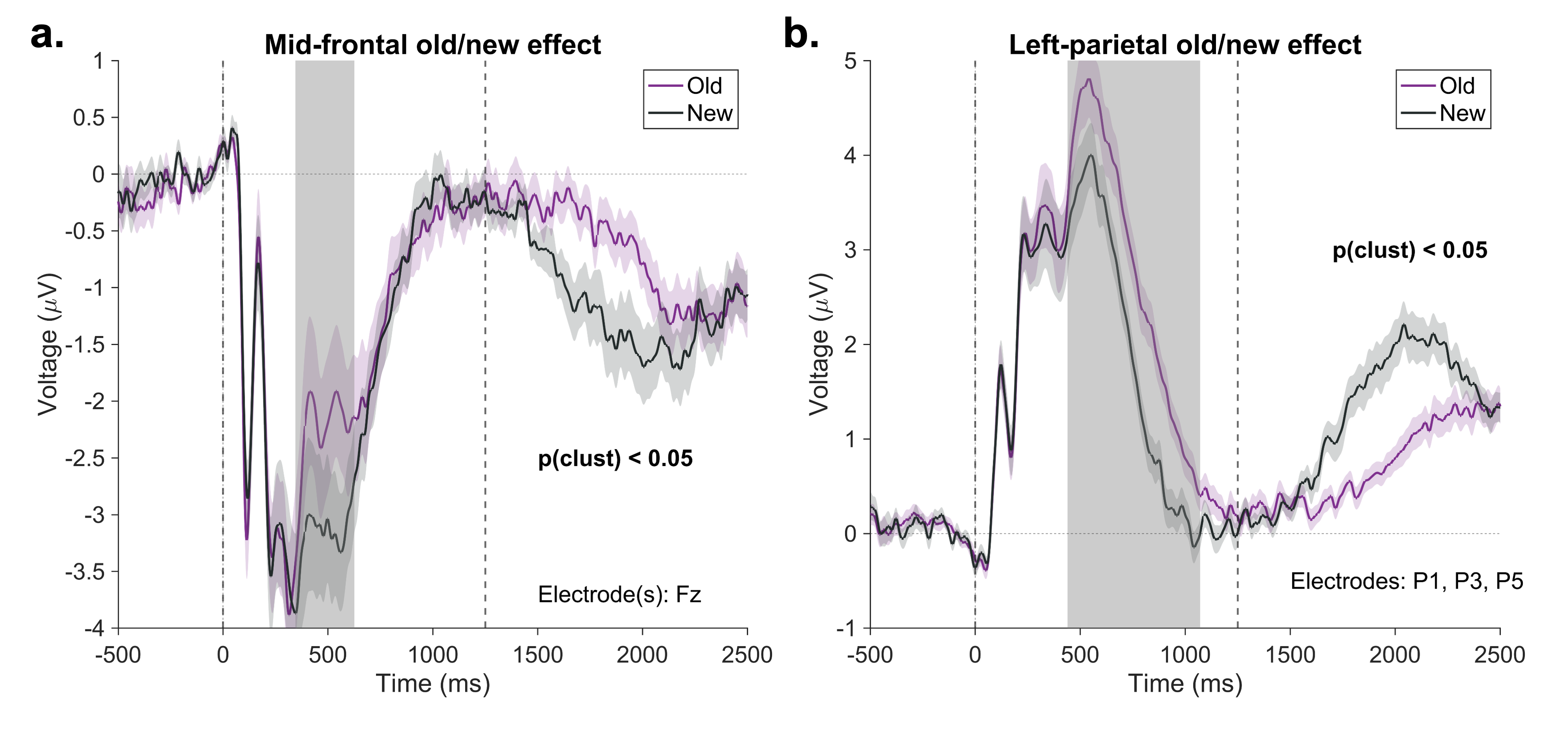

Supplement: Supplementary file 1 — Supplementary Information 1. [file 41598_2022_14090_MOESM1_ESM.png]

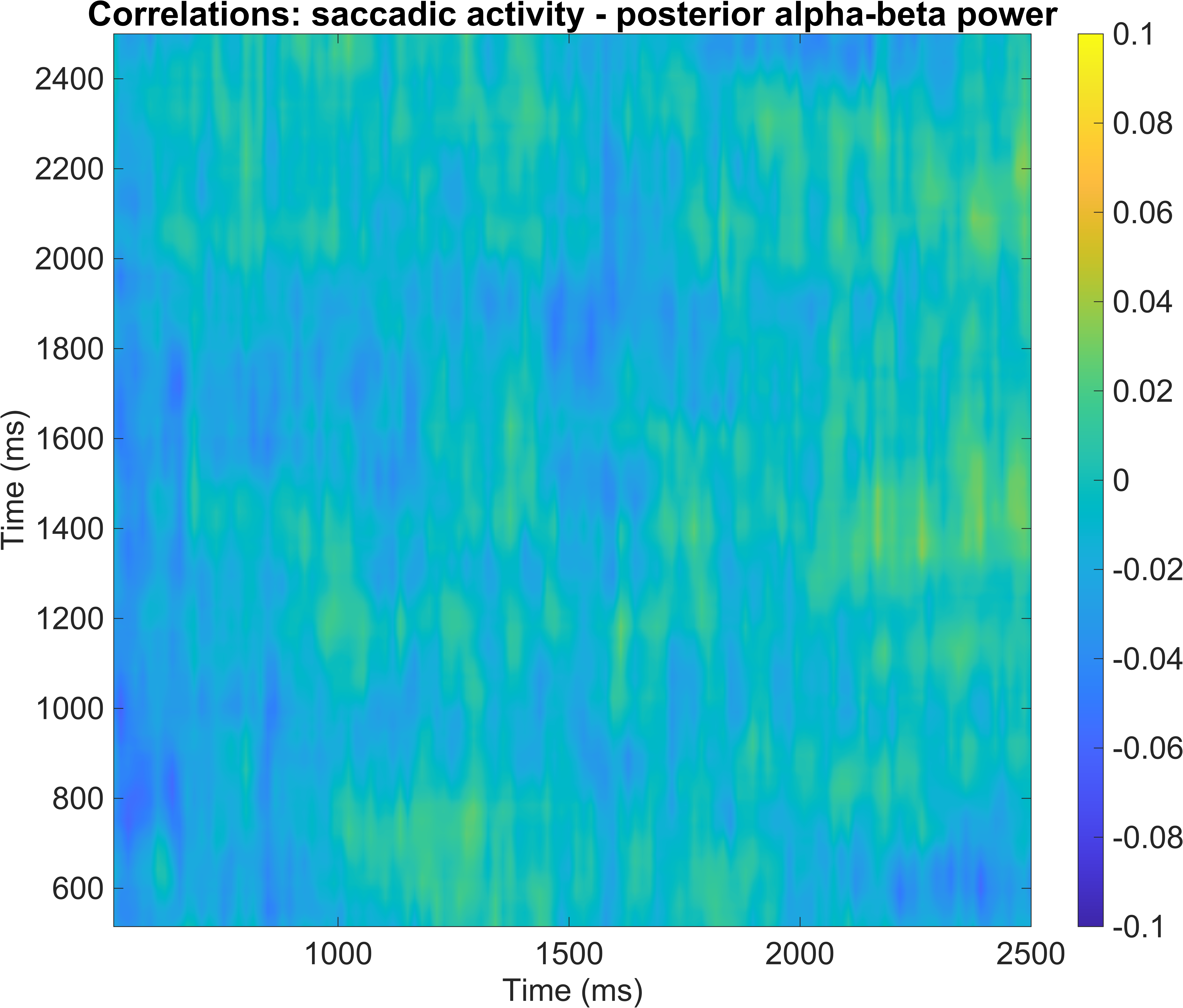

Supplement: Supplementary file 2 — Supplementary Information 2. [file 41598_2022_14090_MOESM2_ESM.png]
