# Supplementary material for: Pattern reinstatement and attentional control overlap during episodic long-term memory retrieval
Source: Sci Rep. 2022 Jun 24;12:10739. doi: 10.1038/s41598-022-14090-4 (PMC9232640; doi:10.1038/s41598-022-14090-4)
Supplement: Supplementary file 3 — Supplementary Information 3. [file 41598_2022_14090_MOESM3_ESM.docx]

**Supplementary materials**

**Pattern reinstatement and attentional control overlap during episodic long-term memory retrieval**

1. **ERP analysis: old-new effect**

In order to investigate the brain activity differentially elicited for old vs. new items presented during retrieval, we conducted an ERP analysis, focusing on the mid-frontal- and left-parietal old/new effect^1^. For the current analysis we used the same preprocessing pipeline described in the Methods section, with the following differences: (i) trials containing new objects were also included in the analysis; (ii) we did not consider correctness or any cutoff reaction time as a trial inclusion criterion. In order to obtain the midfrontal and left-parietal ERPs, the activity associated with old and new trials was averaged separately over the channels of interest (mid-frontal ERP: Fz; left-parietal ERP: P1, P3, P5). Channel selection was based on previous studies investigating these old/new ERP effects^2,3^. In order to correct for multiple comparisons, we conducted the same cluster-based permutation procedure described in the Methods section, restricted to the time window 0-1250 ms (see Methods).

As shown in Figure S1, the cluster-based permutation procedure revealed that both the midfrontal as well as the left-parietal activity associated with the presentation of old vs. new objects significantly differed. For the former analysis, we obtained a significant cluster between 345-626 ms, while for the latter analysis, the significant cluster ranged between 440-1071 ms, thus successfully replicating previous findings^1–3^.

1. **Saccadic activity and alpha-beta lateralization during the encoding phase**

In order to investigate whether saccades had any effect on lateralized alpha modulations during the encoding phase, we correlated single trial saccade-related activity (i.e., ERPs measured at channels F9/10) with single trial lateralized alpha-beta power (both with respect to the imagination position). As such, saccadic modulations were obtained by calculating the contralateral-minus-ipsilateral activity with respect to the left and right imagination position (i.e., for the left condition: the activity of F10 minus F9; for the right condition: the activity of F9 minus F10). A similar procedure was adopted for the alpha-beta oscillations but using the posterior cluster of electrodes: PO7/8, P7/8, P5/6, TP7/8. In addition, we adopted a single-trial pre-stimulus baseline, following the recommendations of Grandchamp and colleagues^4^. Once the activity corresponding to the left and right conditions was calculated, data were averaged over the posterior cluster and the frequencies-of-interest (i.e., 8 -20 Hz). Since the object offset corresponding to the beginning of the imagination task was set to 500 ms after object presentation, the time window for the current analysis was restricted to 500-2582 ms. Subsequently, for each of the two imagination conditions, Spearman correlations between frontal and posterior contralateral-minus-ipsilateral activity were calculated across all time points (i.e., the activity of all time points was correlated with the activity of all other time points). Finally, the obtained correlation coefficients were fisher z-transformed and averaged for the two conditions, thus obtaining a matrix of timepoint x timepoint x participant. In order to test statistical significance, the resulting fisher-z transformed correlation values were contrasted against zero in a cluster-based permutation procedure. We adopted a similar analysis to the one described in the Methods section, with the exception that in each iteration the fisher z-transformed values were randomly exchanged with matrices containing zeros (instead of re-shuffling the condition labels). The procedure did not reveal any significant cluster (figure S2). This shows that we were not able to establish a reliable link between alpha-beta power and saccadic activity at a single trial level.

**References**

1. Rugg, M. D. & Curran, T. Event-related potentials and recognition memory. *Trends Cogn. Sci.* **11**, 251–257 (2007).

2. MacLeod, C. A. & Donaldson, D. I. Investigating the Functional Utility of the Left Parietal ERP Old/New Effect: Brain Activity Predicts within But Not between Participant Variance in Episodic Recollection. *Frontiers in Human Neuroscience* vol. 11 (2017).

3. Danker, J. F. *et al.* Characterizing the ERP Old-New effect in a short-term memory task. *Psychophysiology* **45**, 784–793 (2008).

4. Grandchamp, R. & Delorme, A. Single-Trial Normalization for Event-Related Spectral Decomposition Reduces Sensitivity to Noisy Trials. *Frontiers in Psychology* vol. 2 (2011).
